# Examining evidence for neighbourhood variation in the duration of untreated psychosis

**DOI:** 10.1016/j.healthplace.2009.09.013

**Published:** 2010-03

**Authors:** J.B. Kirkbride, D.J. Lunn, C. Morgan, J.M. Lappin, P. Dazzan, K. Morgan, P. Fearon, R.M. Murray, P.B. Jones

**Affiliations:** a Department of Psychiatry, University of Cambridge, Box 189, Level E4, Addenbrooke's Hospital, Hills Road, Cambridge CB2 0QQ, UK; b MRC Biostatistics Unit, Institute of Public Health, Forvie Site, Robinson Way, Cambridge CB2 0SR, UK; c Psychological Medicine & Psychiatry, Institute of Psychiatry, de Crespigny Park, London SE5 8AF, UK

**Keywords:** Neighbourhood, DUP, Spatial epidemiology, Psychosis, Geography

## Abstract

**Background:**

Family involvement in help-seeking is associated with a shorter duration of untreated psychoses [DUP], but it is unknown whether neighbourhood-level factors are also important.

**Methods:**

DUP was estimated for all cases of first-episode psychoses identified over 2 years in 33 Southeast London neighbourhoods (*n*=329). DUP was positively skewed and transformed to the natural logarithm scale. We fitted various hierarchical models, adopting different assumptions with regard to spatial variability of DUP, to assess whether there was evidence of neighbourhood heterogeneity in DUP, having accounted for *a priori* individual-level confounders.

**Results:**

Neighbourhood-level variation in DUP was negligible compared to overall variability. A non-hierarchical model with age, sex and ethnicity covariates, but without area-level random effects, provided the best fit to the data.

**Discussion:**

Neighbourhood factors do not appear to be associated with DUP, suggesting its predictors lie at individual and family levels. Our results inform mental healthcare planning, suggesting that in one urbanised area of Southeast London, where you live does not affect duration of untreated psychosis.

## 1 Introduction

Emerging evidence from social epidemiology suggests that the incidence of schizophrenia and other non-affective psychoses varies spatially (March et al., 2008), implicating societal-level stressors in their aetiology. Importantly, the relationship between urbanicity and schizophrenia risk has been shown to extend back as far as birth (Mortensen et al., 1999), making reverse causality – social drift – insufficient to explain this variation alone. Many of these societal-level factors appear to be related to an absence of social cohesion or support (Allardyce and Boydell, 2006). Thus, people of ethnic minority status have a greater risk of psychosis when they are more isolated from other ethnic minority groups (Boydell et al., 2001), live in more fragmented residential patterns (Kirkbride et al., 2007b) or are exposed to greater levels of perceived discrimination (Veling et al., 2007). Neighbourhoods with greater levels of residential social mobility (Silver et al., 2002) and other indexes of social fragmentation (Allardyce et al., 2005) and isolation (van Os et al., 2000) have been associated with higher rates of schizophrenia, a finding that persists after adjustment for individual-level sociodemographic factors and the level of socioeconomic deprivation at the neighbourhood level (Kirkbride et al., 2007a, 2008).

However, whether other aspects of psychotic disorders, such as the duration of untreated psychosis [DUP], are also associated with societal-level factors is not known. One recent study has shown that the probability of hospital admission for a psychotic disorder was associated with neighbourhood-level informal social control (Drukker et al., 2006)—defined as the “willingness of local residents to intervene for the common good…–...depends in large part on conditions of mutual trust and solidarity among neighbours”. (Sampson et al., 1997, p. 919) This raises the possibility that DUP may also be associated with neighbourhood-level social factors, since people exhibiting deviant behaviour may come to the attention of services more quickly in socially cohesive areas. Understanding whether DUP is associated with factors at the societal level has potentially important implications for mental healthcare planning and possible strategies to reduce the length of time subjects are without treatment. We therefore tested whether DUP varied at the neighbourhood level, after taking into account known individual-level risk factors known to be associated with DUP (Morgan et al., 2006a).

## 2 Methodology

All subjects presenting to the Southeast London centre of the Aetiology and Ethnicity in Schizophrenia and Other Psychoses [ÆSOP] study, over a 2-year period, with a suspected first-episode psychosis were included in the current study. The ÆSOP study is a large, three-centre study of first-episode psychoses conducted between 1997 and 1999, for which a detailed methodology has previously been given (Kirkbride et al., 2006). For brevity, the methodology below is restricted to specific features relevant to the hypotheses delineated above.

### 2.1 Case ascertainment

All subjects aged 16–64 years old and presenting to services with a suspected first-episode psychosis [FEP], resident in one of 33 neighbourhoods in Southeast London between September 1997 and August 1999 were included in the study. Subjects underwent a battery of assessments, including the Schedules for Clinical Assessment in Neuropsychiatry [SCAN], Personal and Psychiatric History Schedule [PPHS] and a sociodemographic schedule. Ethnicity was rated using all available information, including self-report. For the purposes of this analysis we collapsed ethnicity into a seven-category variable (white British, other white, black Caribbean, black African, Asian (Indian subcontinent), mixed ethnicity, other ethnic groups). Subjects were excluded if they were found to have an organic basis to their disorder. A panel of clinicians, who were blinded to the ethnicity of the subject by the clinician presenting the case, made consensus ICD-10 diagnoses [F10-33]. Inter-rater reliability was high (Kirkbride et al., 2006).

### 2.2 Duration of untreated psychoses

Data relating to date of onset of psychosis were collated from interviews with the patient and a close relative of the patient, and from clinical notes using the World Health Organization [WHO] Personal and Psychiatric History Schedule [PPHS] (WHO, 1996). DUP was defined as the period in days from the onset of psychosis to first contact with statutory mental health services. In line with previous studies (Craig et al., 2000), onset of psychosis was defined as the presence for 7 days or more of one of the following psychotic symptoms: delusions; hallucinations; marked thought disorder; marked psychomotor disorder; and bizarre, grossly inappropriate and/or disorganised behaviour with a marked deterioration in function. A rating of onset was made only when there was a clear, unequivocal description from any source of symptoms meeting these criteria. Previous studies have used a number of different end-points in defining DUP, including first admission (Craig et al., 2000), and start of antipsychotic medication (Norman and Malla, 2001). For our study, patients were included whether they were admitted to hospital or treated in the community, and not all were prescribed antipsychotic medication within the time psychotic frame of the study. Our end-point, therefore, was contact with mental health services. Inter-rater reliability was assessed for the authors who rated DUP (CM, RA, JML) by each independently rating DUP on a random subset of 50 participants, and found to be satisfactory (*r*=0.903).

### 2.3 Statistical analyses

**Fig. 1:**
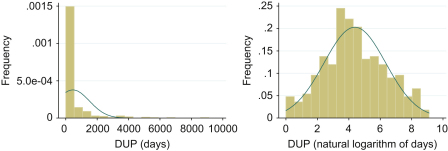
Distribution of DUP in days (a) and after log transformation (b).

We adopted a hierarchical modelling approach (Goldstein et al., 2002) to test our hypotheses since it allows us to take into account the natural structure of the data, i.e. individuals nested within neighbourhoods, and also to examine whether the distribution of DUP is spatially patterned. Here we used 32 of 33 electoral wards (*N*∼6000) in Southeast London as our neighbourhood unit of analysis (one ward was ignored due to there being no incident cases) (Fig. 1).

DUP was highly skewed (see Fig. 1a) with a median length of 69.5 days (9.9 weeks) (inter-quartile range: 22.5–314.0). Taking its natural logarithm gave a satisfactory approximation to the normal distribution (Fig. 1b). In our model, we let *T*_*ij*_ denote the natural logarithm of DUP for the *j*th individual in ward *i* $\left(i=1,...,K=32;\;j=1,...,n_{i},\;\sum_{i=1}^{K}n_{i}=314\right)$. For all models we then have

$$T_{ij}=\mu_{ij}+e_{ij},$$

where $\mu_{ij}$ denotes a regression equation (see below) and $e_{ij}$ is a normally distributed residual term (with mean zero and variance $\sigma_{e}^{2}$) that accounts for any variability not explained by the regression equation. To address the questions above, we developed six models, each with a different form chosen for $\mu_{ij}$. Our first model was an empty model involving only unstructured, area-level random effects. Model 2 introduced age (continuous), sex (dichotomous) and ethnicity (7 categories) as individual-level covariates to this model. Ethnicity was fitted as six dummy variables, each coded 0/1 for one of the black and minority ethnic groups under study. The dummy variable for the baseline white British group was omitted (for identifiability of the model). The third model included individual-level covariates but no random effects at the neighbourhood level, in effect a single-level model. Model 4 was another empty model (without covariates), this time fitted with spatially structured area-level random effects to investigate possible spatial patterning of DUP between neighbourhoods. Our fifth model was an extension of model 4, including individual-level covariates as *a priori* confounders. Our final model included individual-level covariates and both unstructured and spatially structured random effects at the neighbourhood level. If we observed substantial random effects at the neighbourhood level, we went on to consider whether possible socioenvironmental factors between wards (for example social cohesion) explained this variation. Below we provide the specification for our most complex model, model 6, from which all other models can also be derived.

$$\mu_{ij}=\alpha +\beta_{1}x_{ij1}+\cdots +\beta_{8}x_{ij8}+R_{i}+S_{i},$$

where α is an intercept term and $\beta_{k}$ is the coefficient for the *k*th individual-level covariate, whose value for the *j*th individual in ward *i* is denoted $x_{ijk}$. These make up the fixed effects. $R_{i}$ is an additional unstructured random effect for the *i*th ward; we assume the $R_{i}$’s arise from a common, normal ‘population’ distribution with mean zero and unknown standard deviation $\sigma_{R}$:

$$R_{i}∼N(0,\sigma_{R}^{2}),\;i=1,...,K=32.$$

$S_{i}$ is the additional effect of spatially structured random effects in the *i*th ward. We adopted the conditional autoregressive (CAR) model proposed by Besag et al. (1991) to represent any spatially structured variability in the data. This model weights the random effect for a given ward by the random effects in adjacent wards, essentially smoothing the data to take into account any clustering at the neighbourhood level. Spatially structured random effects, $S_{i}$, are also assumed to arise from a normal distribution, but now

$$S_{i}|S_{j},\;j\neq i∼N\left({\bar{S}}_{i},\frac{\sigma_{S}^{2}}{\nu_{i}}\right),\;{\bar{S}}_{i}=\frac{1}{\nu_{i}}\sum_{j\in \delta_{i}}S_{j},$$

where $\delta_{i}$ is the set of neighbours of ward *i*, in this case those wards directly adjacent to *i*, and $\nu_{i}$ is the number of such neighbours.

### 2.4 Implementing hierarchical models

Hierarchical models were fitted using WinBUGS (version 1.4.3) (Lunn et al., 2000) and its spatial modelling extension GeoBUGS (version 1.2) (Thomas et al., 2004). WinBUGS uses Markov chain Monte Carlo (MCMC) simulation (Metropolis et al., 1953; Hastings, 1970; Geman and Geman, 1984; Gelfand and Smith, 1990) to obtain estimates of the *posterior distribution* of model parameters (fixed and random effects). The posterior distribution summarises the uncertainty regarding unknown parameters that remains after combining our *a priori* knowledge (if any) with the evidence contained in the data via Bayes’ theorem (Gelman et al., 1995); in other words, it is the probability distribution of the model parameters given the data (Kirkwood and Sterne, 2003). Both point (median) and interval estimates for parameters of interest are readily obtained from the posterior distribution. The latter are referred to as *credible intervals* and have a direct probabilistic interpretation, unlike confidence intervals; for example, there is a 95% probability that a given parameter lies within its 95% credible interval (conditional on the data and the various modelling assumptions). Choices between different models are based on the Deviance Information Criterion (DIC) (Spiegelhalter et al., 2002), which is a generalisation of AIC (Akaike, 1974) suitable for application to hierarchical models. It comprises a measure of model fit penalised by an appropriate measure of model complexity. A lower DIC indicates a better fitting model.

We choose WinBUGS as our analysis tool primarily for the flexibility that it offers in terms of modelling assumptions (Lunn et al., 2009). The software makes use of so-called *Bayesian* ideas, as described above, which, to the best of our knowledge, are necessary for straightforward implementation of the required CAR model (Besag et al., 1991) in freely available software. Bayesian methods also allow the specification of *a priori* knowledge regarding the unknown parameters, e.g. *σ*_*R*_, *σ*_*S*_, expressed in the form of a ‘prior’ probability distribution. However, in cases such as this, where we have no such prior knowledge, we can typically specify a ‘minimally informative’ prior distribution such that our inferences are based entirely on the likelihood function, as in a more conventional analysis.

## 3 Results

Three-hundred and twenty-nine subjects presented to services in the Southeast London study area of the ÆSOP study over 2 years. Of these, 15 had to be excluded from this analysis because they were of no fixed abode, or their address at first presentation could not be otherwise established. Excluded subjects were more likely to be men (Chi^2^ test on one degree of freedom: 5.7; *p*=0.02) but did not differ from the remainder of the sample in terms of age (*p*=0.49), ethnicity (*p*=0.78) or DUP (*p*=0.82). The remaining subjects were distributed across 32 wards with a median number of 8 cases per ward (min: 1, max: 31). Sociodemographic data on the sample and detailed investigation of individual-level predictors of DUP have previously been reported (Kirkbride et al., 2006, 2007a; Morgan et al., 2006a) (Table 1).

The main results from our six models are summarised in Table 1. According to the DIC all models performed better than our empty models with unstructured (Model 1 DIC: 1312.62) and structured (Model 4 DIC: 1314.73) area-level random effects. Overall, however, the best model did not contain any hierarchical random effects (Model 3 DIC: 1281.52), implying that DUP was not associated with neighbourhood-level characteristics. Model 3 is a single-level model fitted with individual-level age, sex and ethnicity. Our other models (2, 4, 5, 6) did allow for variation in DUP between neighbourhoods, and hence provided estimates of the area-level variability. However, when considered as a proportion of the overall variance, these are very small (always less than 2%, see Table 1), and so these models provide further evidence that any variation in DUP at the neighbourhood level is unlikely to be meaningful from a clinical or public health perspective. As previously reported (Morgan et al., 2006a) there was no evidence of a difference in DUP between men and women, but as before, we observed that longer DUP was significantly associated with increased age in this subset of our previous sample. There was little suggestion that the length of DUP varied between ethnic groups, although DUP for black African subjects was significantly shorter than for the white British group, again, as previously reported (Morgan et al., 2006b).

**Fig. 2:**
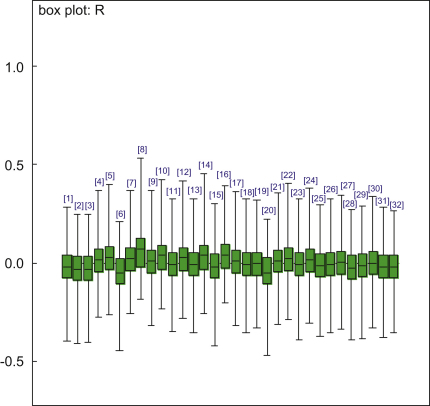
Box plot of random effects at neighbourhood level (model 2).

Fig. 2 provides further illustration as to why area-level random effects are an unimportant addition to the model. The figure shows a summary of the posterior distribution for each random effect *R*_*i*_ from Model 2 (these are estimated automatically as part of the Bayesian/MCMC analysis). All of the posteriors are similar, indicating a lack of heterogeneity across areas, and not one random effect differs significantly from zero. In addition, all of them have point estimates (posterior medians) very close to zero in comparison with the scale of the residual variation ($\sigma_{e}$∼1.9 from Table 1). The considerable uncertainty regarding the values of these ward-level effects, reflected in the size of the plotted credible intervals, inflated our estimate of the ward-level variation above what it would be by considering point estimates alone, to $\sigma_{R}^{2}$∼0.013 (Model 2). As can be seen from Table 1, however, this represented only 0.36% of the total variance, which was insufficient compensation for including 32 random effects and their standard deviation as model parameters (although these collectively only used up ∼2 ‘degrees of freedom’ – because the random effects were very similar – according to the mathematical theory underpinning DIC (Spiegelhalter et al., 2002)). Indeed, the ‘model fit’ component of DIC was almost identical between Models 2 and 3. As our models suggested that any ward-level random effects were small, we were unable to identify any spatial structure among them.

We repeated the above analyses separately for the non-affective psychoses (ICD-10 F20–29) and affective psychoses (F30–33), but found no evidence that variation in DUP could be attributed to the neighbourhood level for either outcome (Fig. 2).

## 4 Conclusion

### 4.1 Principal findings

In a large sample of people with a first episode of any clinically relevant psychosis, we did not find any evidence that variation in the duration of untreated psychoses could be attributed to factors associated with neighbourhood-level characteristics. To the authors’ knowledge, this study is the first to have tested this hypothesis. Our results should provide useful information for mental healthcare planners as they suggest that in one urbanised area of Southeast London, where you live does not have an effect on the duration you went without contact with services for a first episode of psychosis, though important variation in absolute rates of schizophrenia remain (Kirkbride et al., 2007a). Efforts to reduce the duration of untreated psychosis should focus on those individual-level factors previously shown to be associated with DUP (Morgan et al., 2006a), including mode of onset, diagnosis, unemployment and family involvement with referral.

### 4.2 Strengths and limitations of the study

Our study has a number of strengths. Subjects were obtained from one geographically well-defined area of the ÆSOP study, an epidemiologically complete study of all subjects presenting with a first-episode psychosis over a 2-year period. The study included a leakage study to minimise the possibility that subjects may have been missed by the original screen. The study is known to provide accurate estimates of incidence and other epidemiological markers (Kirkbride et al., 2006), and has previously provided robust findings regarding clinical and social determinants of DUP (Morgan et al., 2006a). In this paper, we did not model all clinical and social determinants of DUP because we were able to reject our alternative hypothesis of variation in DUP at the neighbourhood level, having included only a small subset of sociodemographic factors. Unlike other studies of DUP (Norman and Malla, 2001), we were able to include non-hospitalised subjects in our study to minimise selection bias, by using a broad definition of DUP as contact with services, including treatment in the community and patients who were not prescribed antipsychotics during the study period.

To rate DUP we used all available information from interviews with patients and relatives and from case records; for a proportion of patients, the only available information was from case records. All patients were included in our analyses. We made a series of comparisons between patients for whom we had key informant data and those for whom we did not, to assess whether there was any notable difference between them and to assess whether there was any evidence of systematic information bias. There was no evidence of any difference between the groups; importantly, there was no evidence of a systematic difference in estimates of DUP (Morgan et al., 2006a). Furthermore, we were not able to investigate the role of substance use (or indeed other possible factors such as stigma and beliefs about mental illness and mental health services) in determining DUP, as these data were not appropriately recorded for this study. This is a limitation that needs to be considered in future research.

**Fig. 3:**
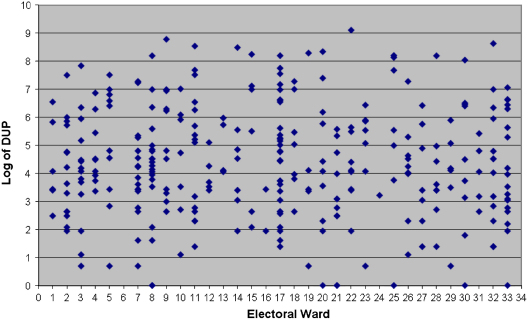
Distribution of individual DUP (log) by electoral ward.

It is always possible that our null results are due to a lack of statistical power to detect variation in DUP in our sample. However, using a near identical sample in Southeast London and hierarchical modelling, we have previously demonstrated neighbourhood-level variation in *incidence rates* (Kirkbride et al., 2007a). In any case, there is enormous overlap in the range of DUPs between wards, as demonstrated in Fig. 3. Thus any area-level variation that may exist is likely to have been dwarfed by the residual (individual-level) variation. Even if we were able to detect any such variation, the signal-to-noise ratio would be so low as to render any further investigation of limited value. Furthermore, our empty model with spatially structured effects (model 4) indicated that the absence of variation in DUP at area level could not be explained by the possibility that individual-level covariates masked important neighbourhood-level variance (i.e. model 5) (Schwartz, 1994). In addition, further analyses conducted on the same data (results not shown here) have demonstrated that none of the area-level factors observed in our data set are significant predictors of DUP (Fig. 3).

### 4.3 Meaning of the findings

For the first time, whether variation in DUP is associated with any neighbourhood-level characteristics has been investigated. That where you live does not appear to affect the length of time without treatment for severe mental illness is encouraging for mental health service providers in urbanised communities. Our study therefore supports individual- and familial-based initiatives to reduce the duration of untreated psychosis in the community, such as the provision of early intervention in psychosis services, which aim to improve the long-term outcomes of severe mental illness by providing early treatment and care to people with psychosis (Department of Health, 2001; Lester et al., 2009) It is, however, noteworthy that important clinical and social inequalities in DUP (Morgan et al., 2006a), and pathways to care (Morgan et al., 2005a, 2005b), remain for some groups. There is also some evidence that poor social support may lengthen the duration of untreated psychosis (Peralta et al., 2005), which overlaps with our previous finding that family involvement is also important in reducing DUP (Morgan et al., 2006a). One previous study has shown that DUP can be reduced for individuals when early detection services exist in their communities (Melle et al., 2004), suggesting that while the determinants of DUP may not operate directly at the neighbourhood level, it does provide a suitable target for intervention strategies to reduce DUP.

We would discourage generalising our findings on DUP and neighbourhood to other, less urban communities, where genuine geographical barriers to accessing mental health services may exist, potentially resulting in neighbourhood-level differences in DUP. This may be particularly true in rural communities where less is known about the effectiveness of early intervention services and effects on DUP (Welch and Welch, 2007). Our findings relate to a relatively small, unique and extremely densely populated area of inner-city London and do not betray possible variation in DUP at other spatial scales. For example, one recent study has found evidence of an inverse relationship between DUP and national gross domestic product of low and middle income countries, suggesting that broader socioeconomic forces at higher (national) geographical levels may be important in predicting DUP (Large et al., 2008).

Our results also have implications for understanding an association we have recently reported between social cohesion and the incidence of schizophrenia in the same community (Kirkbride et al., 2008). Our data suggested that the relationship between social cohesion and schizophrenia incidence was u-shaped, such that the neighbourhoods with both the lowest and highest levels of social cohesion had elevated rates of schizophrenia. We proposed two hypotheses to explain the higher rates in neighbourhoods with higher social cohesion: first that this may be due to higher levels of informal social control (Drukker et al., 2006), such that people exhibiting deviant behaviour in these communities came to the attention of services quicker than in other areas, resulting in improved detection and artificially raised incidence rates. If this were true, then one would have expected to have observed shorter DUP in areas with high informal social control, but the present study did not observe important variation in DUP at the neighbourhood level. Since we did not, we can return to the second hypothesis we originally proposed: that social cohesion may operate contextually to govern the risk of psychosis (Kirkbride et al., 2008). That is to say, for a given individual, living in a community with high social cohesion may increase the risk of psychosis to a greater extent than living in a community with absolutely low rates of social cohesion, if that individual is unable to access the social cohesion perceived to be available in their community. This effect is akin to the ethnic density hypothesis (Boydell et al., 2001) and is consistent with the finding by van Os et al. (2000), which showed that the risk of schizophrenia for single people increased as the proportion of single people in the community decreased (and thus perceived social isolation increased). This hypothesis should now be tested directly.

By investigating the possible association between DUP and neighbourhood-level characteristics we have addressed a previously untested hypothesis. Our results should provide some useful information for mental healthcare planners in urban areas, though further research in different settings will be needed to validate our findings elsewhere.

## Declaration of Interest

None declared.

## Figures and Tables

**Table 1:** Results of hierarchical modelling of DUP by electoral ward for all clinically relevant psychoses.^a^

	**Model 1 (unstructured random effects)**	**Model 2 (model 1+age, sex and ethnicity)**	**Model 3 (age, sex and ethnicity only—single-level model)**	**Model 4 (spatially structured random effects)**	**Model 5 (model 4+age, sex and ethnicity)**	**Model 6 (Model 5+unstructured random effects)**
**DIC**	1312.62	1283.22	1281.52	1314.73	1285.49	1287.62
$\sigma_{e}$	2.0 (1.8, 2.1)	1.9 (1.7, 2.0)	1.9 (1.7, 2.2)	2.0 (1.8, 2.1)	1.9 (1.7, 2.0)	1.9 (1.7, 2.0)
$\sigma_{R}$	0.14 (0.0055, 0.44)	0.11 (0.0017, 0.38)	NA	NA	NA	0.12 (0.00028, 0.40)
$\sigma_{S}$	NA	NA	NA	0.36 (0.13, 0.81)	0.21 (0.068, 0.43)	0.19 (0.056, 0.40)

**Proportion of variance attributable to area-level random effects** (%)	0.51 (0.00078, 4.7)	0.36 (0.000078, 4.1)	NA	1.3 (0.20, 5.1)	1.3 (0.13, 5.2)	1.8 (0.24, 6.6)

**Age** (years)	NA	0.052 (0.032, 0.072)*	0.052 (0.032, 0.072)*	NA	0.053 (0.033, 0.073)*	0.053 (0.032, 0.072)*
				
**Sex** (men vs. women)	NA	0.24 (−0.17, 0.67)	0.24 (−0.18, 0.66)	NA	0.24 (−0.17, 0.66)	0.24 (−0.18, 0.67)

**Ethnicity**						
White other	NA	−0.13 (−0.93, 0.68)	−0.13 (−0.92, 0.68)	NA	−0.13 (−0.92, 0.69)	−0.13 (−0.93, 0.67)
Black Caribbean	NA	0.20 (−0.34, 0.76)	0.21 (−0.34, 0.75)	NA	0.21 (−0.34, 0.76)	0.21 (−0.33, 0.77)
Black African	NA	−0.74 (−1.4, −0.10)*	−0.73 (−1.4, −0.09)*	NA	−0.73 (−1.4, −0.10)*	−0.74 (−1.4, -0.10)*
Asian	NA	−1.2 (−3.1, 0.68)	−1.2 (−3.1, 0.65)	NA	−1.2 (−3.1, 0.71)	−1.2 (−3.1, 0.68)
Mixed ethnicities	NA	−0.86 (−1.9, 0.14)	−0.89 (−1.9, 0.09)	NA	−0.85 (−1.9, 0.15)	−0.83 (−1.8, 0.17)
Other ethnicities	NA	−0.40 (−1.6, 0.77)	−0.41 (−1.6, 0.78)	NA	−0.41 (−1.6, 0.79)	−0.43 (−1.6, 0.76)

a All figures rounded to 2 significant figures. Numbers in brackets indicate 95% credible intervals. Coefficients represent the change in expected log_e_-DUP associated with a unit increase in the relevant covariate (i.e. age in years, men vs. women and white British vs. other ethnic groups).

* Statistically significant, in the sense that the 95% credible interval excludes zero.
